# Isoeugenol has a non-disruptive detergent-like mechanism of action

**DOI:** 10.3389/fmicb.2015.00754

**Published:** 2015-07-28

**Authors:** Morten Hyldgaard, Tina Mygind, Roxana Piotrowska, Morten Foss, Rikke L. Meyer

**Affiliations:** ^1^Interdisciplinary Nanoscience Center, Aarhus UniversityAarhus, Denmark; ^2^Antimicrobials and Antioxidants, Nutrition and Health, DuPont Nutrition BiosciencesBrabrand, Denmark; ^3^Department of Bioscience, Aarhus UniversityAarhus, Denmark

**Keywords:** isoeugenol, antimicrobial, phenylpropene, mechanism of action, essential oil

## Abstract

Isoeugenol is an essential oil constituent of nutmeg, clove, and cinnamon. Despite isoeugenol's promising antimicrobial activity, no studies have yet investigated its mode of antibacterial action at the molecular level. The aim of this study is to clarify isoeugenol's antibacterial mode of action using the Gram-negative and Gram-positive model organisms *Escherichia coli* and *Listeria innocua*, respectively. We determined the antimicrobial activity of isoeugenol against the model organisms, and examined how isoeugenol affects cell morphology, cell membrane permeabilization, and how isoeugenol interacts with phospholipid membranes using vesicle and supported lipid bilayer models. Isoeugenol demonstrated a bactericidal activity against *E. coli* and *L. innocua* that did not affect cell morphology, although the cell membrane was permeabilized. We hypothesized that the cell membrane was the primary site of action, and studied this interaction in further detail using purified membrane model systems. Isoeugenol's permeabilization of calcein-encapsulated vesicles was concentration dependent, and isoeugenol's interaction with giant unilamellar vesicles indicated increased membrane fluidity and a non-disruptive permeabilization mechanism. This contradicted membrane fluidity measurements on supported lipid bilayers (SLBs), which indicated decreased membrane fluidity. However, further investigations demonstrated that the interaction between isoeugenol and bilayers was reversible, and caused membranes to display heterogeneous topography, an increased mass, and a higher degree of hydration. In conclusion, we propose that isoeugenol interacts with membranes in a reversible non-disruptive detergent-like manner, which causes membrane destabilization. Furthermore, we argue that isoeugenol increases membrane fluidity. Our work contributes to the understanding of how essential oil constituents interact with cell components.

## Introduction

Essential oils from plants, herbs, or spices constitute a broad range of low molecular weight organic compounds. These constituents can be categorized into four groups based on their chemical structure: terpenes, terpenoids, phenylpropenes, and a group containing other metabolic degradation products from plants (Hyldgaard et al., 2012a). These oils and their individual constituents have received attention as a natural resource of food additives that can function as aromatic flavorings, antioxidants, and antimicrobials (Rajakumar and Rao, 1993; Brenes and Roura, 2010; Hyldgaard et al., 2012a).

**Figure 1:**
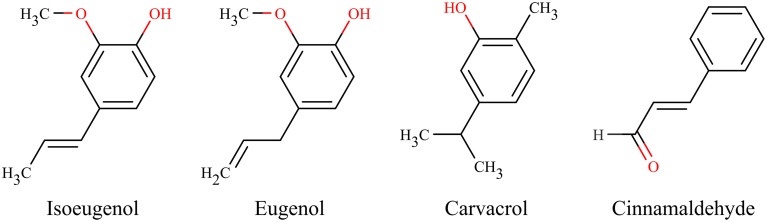
**Chemical structures of essential oil components**.

Isoeugenol belongs to the group of phenylpropenes and occurs naturally in nutmeg, cinnamon, and clove. Interestingly, isoeugenol has proven antimicrobial activity comparable to, and even exceeding, its highly antimicrobial isoform eugenol (Zemek et al., 1979, 1987; Janssens et al., 1990; Laekeman et al., 1990; Dal Pozzo et al., 2012). A general ranking of the antimicrobial activity of essential oil constituents placed eugenol as having higher activity than e.g., carvacrol, cinnamic acid, and cinnamaldehyde (Burt, 2004). The antibacterial activity of isoeugenol has been proposed to be due to its free hydroxyl group and its position of double bonds in α, β positions of the side chain, and a methyl group in the γ position (Figure 1) (Zemek et al., 1979; Laekeman et al., 1990). Isoeugenol's antibacterial activity covers a broad range of Gram-positive and Gram-negative bacteria, including *Escherichia coli, Bacillus licheniformis, Micrococcus luteus, Pseudomonas aeruginosa, Salmonella* type B, and *Staphylococcus aureus* (Zemek et al., 1979, 1987; Laekeman et al., 1990).

In recent years there has been a growing interest in investigating antimicrobial compounds regarding their respective modes of action and categorize these in order to systematize their fields, understand resistance and tolerance mechanisms of microorganisms to antimicrobials, or find alternative treatment options (Yeaman and Yount, 2003; Desbois and Smith, 2010; Hyldgaard et al., 2012a). In spite of isoeugenol's promising antibacterial activity, no studies have yet been performed to identify how isoeugenol affects bacteria at the molecular scale. One study evaluated isoeugenol's mode of antifungal action against *Candida* spp., and suggested that isoeugenol inhibits H^+^-ATPase, which triggers intracellular acidification and cell membrane breakages (Bhatia et al., 2012). Focusing on the mode of action of the structurally closely related compound eugenol, studies indicate that the cell structure becomes distorted due to a non-specific cytoplasmic membrane permeabilization effect together with the ability of eugenol to inhibit enzyme or protein functionality (Thoroski et al., 1989; Wendakoon and Morihiko, 1995; Gill and Holley, 2006b,a; Hemaiswarya and Doble, 2009). Based on these mode of action studies on the related eugenol molecule and isoeugenol's structure, we hypothesize that the hydrophobic isoeugenol inserts into the polar head group region of lipid bilayers where it cause alterations and damages to the cell membrane, which could enhance uptake of isoeugenol, leading to effects on protein/enzyme function.

In the present study we aim to investigate isoeugenol's antibacterial mechanism of action using the model organisms *E. coli* and *L. innocua* together with a broad range of model membrane systems to evaluate isoeugenol's action on cell viability, cellular morphology, enzyme activity, and membrane integrity, fluidity, and hydration.

## Materials and methods

### Materials

Food grade isoeugenol >99% pure was purchased from Sigma-Aldrich Chemie (St. Louis, MO, USA). Isoeugenol was mixed with 1% polysorbate 80 (Merck, Hohenbrunn, Germany) prior to being diluted in either 5 mM MES (2-(N-morpholino)ethanesulfonic acid, Sigma-Aldrich Chemie, Steinheim, Germany), 30 g/L tryptic soy broth (TSB, Merck, Darmstadt, Germany), or TSB with 1.5% agar (TSA, Merck, Darmstadt, Germany), all at pH 6.0. Stains used in this study involve calcein disodium salt obtained from Fluka (Buchs, Switzerland) and fluorescein diacetate (FDA) acquired from Merck (Calbiochem, Darmstadt, Germany), 1,6-diphenyl-1,3,5-hexatriene (DPH) bought from Sigma-Aldrich (D208000, St. Louis, MO, USA), and propidium iodide (PI, L7012, L13152), Alexa Fluor 633 hydrazide (A30634), and dextran labeled with Alexa Fluor 488 (D22910) from Molecular Probes (Oregon, USA). We acquired 1,2-dioleoyl-*sn*-glycero-3-phospho-(1′-*rac*-glycerol) (DOPG, 840475P), 1,2-dioleoyl-*sn*-glycero-3-phosphocholine (DOPC, 850375P), and *E. coli* polar lipid extract [100600C, 67% phosphatidylethanolamine (PE); 23.2% phosphatidylglycerol (PG); 9.8% cardiolipin (CL)] from Avanti Polar Lipids, Inc. (Alabaster, USA). Triton™ X-100 solution was bought from Sigma (St. Louis, MO).

### Bacterial growth conditions

*Escherichia coli* K12 (LZB035, Blades Biological Ltd., Cowden, UK) and *L. innocua* (DSM 20649, DSMZ, Braunschweig, Germany) were cultured in TSB or TSA at 25°C until having an optical density at 620 nm (OD_620_) between 0.05 and 0.25, representing the exponential growth phase. We harvested cells by centrifugation (5000 × g, 10 min), discarded the supernatant, resuspended the pellet in sterile MES-buffer, TSB, or Ringer's solution (Merck, Darmstadt, Germany), ensured that the OD_620_ was between 0.05 and 0.25 using a photospectrometer (Spectrostar Nano, BMG Labtech, Ortenberg, Germany), and diluted the cell cultures with the appropriate solution to cell densities of 10^6^ or 10^7^ CFU/mL.

### Minimum inhibition concentration determination using a broth microdilution assay

Minimum inhibition concentration values were determined using absorbance measurements of serial dilutions of isoeugenol into TSB in concentrations ranging from 0 to 0.6 mg/mL and 0 to 1.5 mg/mL with or without 5 × 10^5^ CFU/mL of *E. coli* and *L. innocua*, respectively. Triplicate cell cultures were prepared in MES-buffer with final cell densities of 10^7^ CFU/mL. Microtiter plates (No. 260887, NUNC plates, Thermo Scientific) with 96-wells were prepared by adding 100 μL TSB in all wells, then adding isoeugenol in a 1.5-serial dilution series to a final concentration ranging either between 0 to 1.2 or 0 to 3 mg/mL. Then 90 μL TSB and 10 μL cell culture were added. Cell-free controls with isoeugenol, and isoeugenol-free controls with cells were prepared in triplicates. The OD_620_ was monitored sequentially every 20 min, for 24 h at 25°C using a plate reader (Sunrise, Tecan, Männedorf, Switzerland). The OD_620_ from cell-free wells containing isoeugenol concentrations were subtracted from sample wells in order to remove background absorbance from isoeugenol. The MIC value was defined as the first concentration resulting in an average increase of OD_620_ ≤ 5% of the growth controls OD_620_ values.

### Isoeugenol's time-kill activity

Detection of MIC values of isoeugenol only identified growth inhibition, but left out information about isoeugenol's effect on cell viability, and if it acted bactericidal or bacteriostatic. We investigated time dependent viability changes using a modified Miles and Misra method (Miles et al., 1938) to evaluate *E. coli* and *L. innocua* susceptibility to isoeugenol while suspended in TSB. Briefly, triplicate cell suspensions with 10^7^ CFU/mL were prepared in Ringer's solution. Cell suspensions were mixed with TSB amended with isoeugenol in 50 mL Falcon tubes reaching final cell densities of 5 × 10^5^ CFU/mL and final isoeugenol concentrations of 0, ½ × MIC, MIC, and 2× MIC in triplicate. Samples were vortexed prior to serial dilution to 10^−6^ in Ringer's solution at time-points 0, 1, 4, 8, 12, 16, 20, and 24 h of incubation at 25°C. Twenty microliters aliquots from each serial dilution step were transferred to two TSA plates in numbered sections, and incubated at 25°C for 24 h. Colony forming units were counted after incubation.

### Atomic force microscopy imaging of whole cells treated with isoeugenol

We used atomic force microscopy (AFM) to investigate if isoeugenol caused changes in cell morphology. Coverslips coated with adhesive mussel proteins (Cell-TAK™, Cell and Tissue adhesive, Becton Dickinson Biosciences, Temse, Belgium) were used to firmly immobilize bacterial cells so they could withstand the lateral forces inflicted by the cantilever during AFM imaging (Meyer et al., 2010). Briefly, coverslips were cleaned in 70% ethanol for 10 min, rinsed with MilliQ water, and air-dried in upright position. Then a 1 × 1 cm square was drawn with a hydrophobic marker (Pap pen, G. Kisker, Steinfurt, Germany) on each coverslip. Adhesive protein solution (28.5 μL freshly made 0.1 M NaHCO_3_, 0.5 μL 1 M NaOH, and 1 μL Cell-TAK™) was added to the hydrophobic square on each coverslip, air-dried 20 min at room temperature. Finally, excessive proteins were removed by submerging coverslips in MilliQ water. The coverslips were air-dried, and stored for a maximum of 2 weeks at 4°C.

We mixed cell suspensions and isoeugenol stock solutions to have final concentrations of 5 × 10^5^ CFU/mL and 0, ½ × MIC, MIC, or 2× MIC, respectively. Samples were incubated 60 min at 25°C, followed by three washing steps of harvesting (5000 × g, 10 min) and resuspending the pellet in MES-buffer. We immobilized cells to the substrate by applying 100 μL cell suspension to the Cell-TAK™ coating, and let the cells settle for 15 min at room temperature before removing non-adhered cells by rinsing with MES-buffer. Coverslips with adhered cells were air-dried and placed in the JPK sample holder (JPK Instruments, Berlin, Germany) prior to imaging with a JPK NanoWizard® II AFM (JPK Instruments, Berlin, Germany) using intermittent contact mode. Freshly UV-irradiated Si_3_N_4_ cantilevers from Olympus (OMCL-AC160TS, Manheim, Germany) with an apical radius less than 10 nm, a resonance frequency of 300 kHz, and a nominal spring constant of 42 N/m were used for imaging. At least three AFM images of *E. coli* and *L. innocua* cells were acquired for each of the triplicate samples at 512 pixels per line at a scan rate of 0.5 Hz of 4 × 4 and 3 × 3 μm areas, respectively. Images were processed in the JPK Data Processing software (Version 4.2.50) by subtracting a first order polynomial from each scan lines, and we manually selected areas to exclude from the fit if they contained important structures. Lines with noise were removed from images, and regenerated by using average values from adjacent lines.

### Evaluation of cell membrane integrity and esterase activity using flow cytometry

We assessed isoeugenol's effect on membrane permeability and esterase activity using PI and FDA, respectively. Propidium iodide is a membrane impermeable dye that binds to nucleic acids and fluoresce red if the membrane integrity is lost, indicative of dead cells. Fluorescein diacetate is a lipophilic non-fluorescent molecule that readily diffuses across membranes, and within metabolically active cells FDA is hydrolyzed by unspecific esterases to the polar membrane-impermeant fluorescent molecule fluorescein.

For *E. coli* we used a 4× PI work solution (kit L13152) made according to the manufacturer, while *L. innocua* cells were stained with a 1.5 mM PI work solution (kit 7012) made with filter-sterilzed deionized water. The FDA work solution was prepared at a 5 mg/mL concentration in acetone. Stains were stored at −20°C in the dark until use.

Cell suspensions were prepared with a cell density of 10^6^ CFU/mL in MES-buffer. Live cells stained with FDA and heat-killed (85°C, 30 min) cells stained with PI, provided the detection boundaries for cells stained with PI or FDA, respectively. Heat-killed cells and cells treated with a final isoeugenol concentration of 0, ½ × MIC, MIC, and 2× MIC at 5 × 10^5^ CFU/mL in MES-buffer for an hour at 25°C were harvested by centrifugation (5000 × g, 10 min). Cell pellets were resuspended in 1 mL MES-buffer, and hereafter mixed with 20 μL FDA and incubated at 37°C for 30 min to allow intracellular enzymatic conversion to fluorescein. Labeled cells were harvested by centrifugation (5000 × g, 10 min), and resuspended in 200 μL MES-buffer and either 66 μL 4 × PI stain for *E. coli* or 2 μL 1.5 mM PI stock for *L. innocua*. Propidium iodide stained samples were incubated in the dark for 15 min at room temperature, and then immediately transferred to flow cytometer for analysis.

A Gallios flow cytometer (Beckman Coulter, Miami, FL, USA) was used for flow cytometry analysis. All channels were calibrated with FlowCheck Fluorospheres (Beckman Coulter, Miami, FL, USA) prior to sample analysis. Fluorescein and PI were excited using the 488 nm laser line. Green fluorescence signal from fluorescein stained cells were collected in the FL1 channel (525/50), whereas PI signal was detected using FL2 (575/26 nm) and FL3 (620/30 nm) channels. Cell density in each sample was adjusted with MES-buffer so signal from 50 to 1000 cells/s was detected under constant flow rate. Samples and controls were all made in triplicate, and at least 30,000 events were collected for each replica.

Flow cytometry data analysis was performed using Kaluza software (version 1.2). Bacterial populations were gated based on their scattering in forward and side direction to minimize noise. Gated populations were analyzed in dual-parameter contour plots of red and green fluorescence signal intensities to differentiate bacterial populations based on their fluorescence properties. The position of a quadrant dividing negative and positive fluorescence signal histograms were chosen for each fluorophore based on the position of a fluorescence-minus-one control population in the contour plot. Which means that dead cells stained with PI worked as the negative fluorescence signal for fluorescein, and vice versa. Statistical significance of isoeugenol's effect on cell membrane integrity and esterase enzyme activity was assessed using One-Way ANOVA test (*p* < 0.05) to compare means of several groups using Minitab® 16.2.4 (Minitab inc., State College, Pennsylvania).

### Evaluation of membrane permeablization by calcein-leakage assay

We assessed isoeugenol's membrane permeabilization efficacy using calcein encapsulated large unilamellar vesicles (LUVs) with an *E. coli* lipid composition representing the cytoplasmic membrane of Gram-negative bacteria.

Calcein encapsulated LUVs were prepared by evaporating the organic solvent from *E. coli* polar lipid extract in a glass vial under nitrogen gas until a dry lipid film appeared. Removal of residual organic solvent was done by having the lipid film under vacuum in a dessicator for 2 h. Vacuum was liberated and nitrogen was applied to the glass vial. We hydrated the dry lipid film with MES-buffer containing 70 mM calcein to a lipid concentration of 10 mg/mL, and dissolved the lipid film by shaking at 200 rpm and 37°C overnight. The resulting multilamellar vesicle lipid solution was alternated eight times between liquid nitrogen and a 40°C waterbath to form unilamellar vesicles. The vesicles were extruded 24 times through a 19 mm Nucleopore Track-Etched membrane with a pore size of 100 nm (Whatman, Clifton, NJ, USA) using a MiniExtruder with a heating block (Avanti Polar Lipids Inc., Alabaster, Al, USA) equilibrated at 37°C. We loaded 500 μL of the calcein-loaded LUVs on a PD-10 desalting column (GE Healthcare, Buckinghamshire, UK) previously equilibrated in MES-buffer, collected the eluated calcein-LUV fraction, and diluted it to a final lipid concentration of 58.5 μM.

Calcein-leakage induced by isoeugenol was monitored using a Tecan GENios Pro microtiter plate reader (Tecan Group, Männedorf, Switzerland) and 96-well black NUNC optical-bottom microtiter plates (No. 265301, Thermo Scientific, Rochester, NY, USA). We added 200 μL of calcein-LUV solution to all wells and measured the calcein fluorescence baseline (*F*_0_) at 37°C by exciting calcein at 448 nm and detecting emission at 485 nm. Isoeugenol was added in wells to a final concentration range between 0.00002 and 2 mg/mL in duplicate. Wells with untreated calcein-LUVs were included as control. The microtiter plate was incubated at 37°C for an hour before measuring the calcein fluorescence signal (*F*). Five microliters of 1% Triton™ X-100 solution was added to all wells to fully permeabilize all vesicles and the calcein signal was measured again (*F*_max_). The microtiter plate was sealed in between individual measurements to prevent evaporation, and all measurements were conducted until a stable baseline was achieved. The relative amount of released calcein was averaged and calculated as:

$$Calcein\;release\left(\%\right)=\frac{F-F_{0}}{F_{max}-F_{0}}\times 100\%$$

Baseline was subtracted from all data points using an average value of a linear section of the graph.

### Confocal laser scanning microscopy of giant unilamellar vesicles treated with isoeugenol

We wanted to visualize how isoeugenol affected membranes and therefore used confocal laser scanning microscopy to monitor the interaction between giant unilamellar vesicles (GUVs) and isoeugenol in solution.

We prepared the GUV solution using a home-build electroformation chamber as previously described by Vad et al. (2010). A 10 mg/mL DOPG:DOPC (20:80) lipid solution were prepared in chloroform. A 15 μL aliquot of the lipid solution was placed on each of the two platinum electrodes in the electroformation chamber. Chloroform was removed by applying nitrogen gas to the electroformation chamber followed by incubation at 95°C for 5 min, and subsequently filled with 800 μL of a 200 mM sucrose solution containing Alexa Fluor 488 and Alexa Fluor 633 hydrazide in a 1:1 ratio. We connected the platinum electrodes with a function generator (Digimess FG100, Grundig Instruments, Nürnberg, Germany) and applied an alternating current (10 Hz, 1.5 V, sinusoidal wave function) for 90 min while keeping the chamber in the dark and at room temperature in order to form GUVs. The GUVs were gently detached from the electrodes by mixing the solution in the chamber with a pipette. Vesicles and free stain in solution were separated by running the GUV solution over a PD-10 desalting column pre-equilibrated with 200 mM glucose solution. The eluted solution containing GUVs were collected in an eight-chambered borosilicate coverglass (No. 155411, Lab-Tek, Nunc) and diluted in 200 mM glucose to a final volume of 300 μL. Giant unilamellar vesicles were allowed to sediment to the bottom of the coverglass by incubating it overnight at 4°C.

The interaction between isoeugenol and GUVs were observed in solution using a LSM 700 confocal laser scanning microscope from Zeiss (Göttingen, Germany). Laser line 488 and 639 nm were used to excite Alexa^488^ and Alexa^633^, while emission were detected after passing a dichroich beam splitter set at 629 nm and either a SP 640 filter and LP 640 filter, respectively. Triplicate time-series of images of the same field of view were taken sequentially every 4 s using a Plan-Apochromat 40 × /1.3 oil immersion objective. Images were recorded using a pixel dwell of 0.79 μs, an image size of 1024 lines per frame, and two times averaging of each line. Prior to recording we added 5 μL of 10 mg/mL isoeugenol solution prepared in 5 mM MES-buffer outside the field of view, and the imaging started approximately 5 min after injection. Usually no interactions were observed the first 10–15 min after injection due to the slow diffusion of molecules within the viscous glucose solution. The arrival of isoeugenol into the field of view was indicated by increased fluctuation of vesicles and changes in direction of solution flow. Time-series of images were analyzed in the blue edition of Zen lite 2012 (version 1.1.2.0, Carl Zeiss Microscopy GmbH, Germany).

### Evaluation of isoeugenol's effect on supported lipid bilayer topography using AFM

Membrane active compounds might induce changes in the integrity and lipid phases of membranes, and therefore AFM has been a valuable tool to investigate the interplay between antimicrobials and supported membranes in numerous studies (Shaw et al., 2006; Yu et al., 2009; Hyldgaard et al., 2012b; Balhara et al., 2013). Using this technique we could visualize the initial effect of isoeugenol on models of bacterial membranes.

Supported lipid bilayers (SLBs) were prepared from *E. coli* polar lipid vesicles prepared as in the calcein-leakage assay without calcein and with a sonication step instead of extrusion through a pore-membrane. After the freeze-thaw cycle, we prepared a small unilamellar vesicle solution (SUV) by sonicating the lipid solution using a rod sonicator (70 W, 50% of maximal power, 3-mm probe diameter, Sonopuls ultrasonic homogenizers, Bandelin Electronic, Berlin, Germany) for 5 min while submerged in an ice bath.

The SUV solution was added to freshly cleaved mica squares glued to 18-mm circular coverslip in a temperature controlled JPK Biocell holder (JPK Instruments, Berlin, Germany) and mixed with MES-buffer containing CaCl_2_ to final concentrations of 0.3 mg lipid/mL and 20 mM Ca^2+^. The solution was incubated 20 min at 37°C in order to allow the SUVs to bind to the substrate and form a SLB. The SLB was washed at least three times with CaCl_2_-free MES-buffer and suspended in CaCl_2_-free MES-buffer at 37°C. Contact mode imaging of SLBs was performed using CSC38/noAl Si_3_N_4_ cantilevers from Mikromash (Tallin, Estonia) with a spring constant of 0.03 to 0.09 N/m. Images of SLBs were acquired of the untreated membrane, and again after 60 min treatment at 37°C with isoeugenol at final concentrations of 0.008 mg/mL or 0.03 mg/mL. The bilayer was rinsed with CaCl_2_-free MES-buffer five times before recording images of the treated SLB. At least three random 5 × 5 μm areas of each of triplicate samples were recorded. AFM Images were acquired as 512 × 512 pixel images at a scan rate of 0.5 Hz using a setpoint below 250 pN.

### Assessing isoeugenol's effect on membrane fluidity by fluorescence anisotropy

Interaction between isoeugenol and membranes could affect membrane fluidity, and therefore we measured changes in anisotropy of the fluorescent probe DPH when situated inside the hydrophobic core of LUVs made of *E. coli* polar lipid or a 20:80 composition of DOPG:DOPC. The DPH probe inserts in the bilayer core, and its depolarization property is then influenced by the packing of the acyl chains (Trevors, 2003).

The DPH powder was dissolved in acetone to a final concentration of 100 μM and added to the chloroform solution containing *E. coli* polar lipid extract or DOPG:DOPC (20:80) at a molar concentration of 1:300 (DPH:lipid). The LUVs were prepared as for the calcein encapsulated LUVs, except addition of calcein and running the LUV suspension over a PD-10 desalting column. The final lipid concentration was adjusted to 56 μM using 5 mM MES-buffer at pH 6.0. Large unilamellar vesicles of *E. coli* polar lipid extract and DOPG:DOPC were mixed with isoeugenol to a final concentration between 0.008 to 0.03 mg/mL and 0.002 to 0.15 mg/mL, respectively. Samples were incubated in the dark for 5 min at 37°C for *E. coli* polar lipid LUVs, and 25°C for DOPG:DOPC LUVs.

Fluorescence determinations were performed with a LS55 fluorospectrometer (Perkin-Elmer, Waltham, USA) with excitation at 360 nm and emission detection at 430 nm with a 10 nm bandwidth. Anisotropy values representing the dynamics of acyl lipid chains were determined by measuring the polarization ratio of emitted light parallel (*I*_VV_) and perpendicular (*I*_VH_) to a vertical excitation polarizer.

Anisotropy values (*r*) were determined as:

$$r=\frac{\left(I_{VV}-G\cdot I_{VH}\right)}{\left(I_{VV}+2\cdot G\cdot I_{VH}\right)}$$

The grating factor is an intrinsic correction factor for polarization calculated as:

$$G=\frac{I_{HV}}{I_{HH}}$$

All DPH fluorescence determinations were performed in triplicate for *E. coli* polar lipid, and in duplicate for DOPG:DOPC LUVs. Each replicate was measured between three and ten times with an integration time of 5 s.

### Assessing binding and interaction with supported lipid bilayers using quartz crystal microbalance with dissipation monitoring

We hypothesized that isoeugenol interacted with the lipid bilayer to cause changes in membrane stability and fluidity, thus we monitored changes in membrane mass and viscoelastic properties upon exposure to isoeugenol. The quartz crystal microbalance with dissipation (QCM-D) monitors changes in resonance frequency (Δ*f*) and energy dissipation (Δ*D*) of an oscillating quartz crystal, which indicates changes in mass and viscoelastic properties of the membrane, respectively.

QCM-D measurements of membranes were performed using the Q-sense E4 system (Q-sense AB, Västra Frölunda, Sweden) thermostated at 37°C, and 5 MHz sensor crystals with 50 nm Silicon dioxide coating (Q-sense Sensor QSX 303 SiO2,Q-sense AB, Västra Frölunda, Sweden city, country). Sensor crystals were stored in 2% SDS, washed with Milli-Q water, dried in a stream of N_2_ gas, and subjected to UV-ozone treatment for 20 min, washed with Milli-Q water, dried with N_2_ gas, followed by a final UV-ozone treatment for 20 min. The crystals were then rinsed with Milli-Q water and dried with N_2_ prior to placing them in the QCM-D mounting chambers. Changes in resonance frequency and dissipation were measured using Q-soft software (version 3.1.25.604) on four channels at the 3rd, 5th, 7th, 9th, 11th, and 13th overtones of the fundamental resonance frequency.

We prepared a 10 mg/mL DOPG:DOPC (20:80) SUV lipid solution following same procedure as for *E. coli* SUVs described in section Evaluation of Isoeugenol's Effect on Supported Lipid Bilayer Topography using AFM, except the thaw-freeze cycle and the rod sonicator power was set to 20%. Sensor crystals were equilibrated in 5 mM MES-buffer (pH 6.0), and SLBs were formed by first introducing 0.6 mg/mL DOPG:DOPC SUVs in MES-buffer at a flow rate of 0.1 mL/min for approximately 3 min, followed by 5 mM MES-buffer with 20 mM CaCl_2_ at a flow rate of 0.1 mL/min using an Ismatec IPC-N 4 peristaltic pump (Ismatec SA, Glattburg, Switzerland). Excess lipid was removed from the SLBs by flushing with CaCl_2_-free 5 mM MES-buffer injected at 0.1 mL/min, and the system was finally equilibrated in the same buffer without flow. Isoeugenol dissolved in 5 mM MES-buffer was then injected at either 0.008 or 0.03 mg/mL at 0.1 mL/min until complete exchange of chamber content, after which the flow was stopped for 60 min. Subsequent washing was performed with 5 mM MES-buffer at 0.1 mL/min prior to stopping the flow to evaluate the reversibility of the interaction.

Data modeling was carried out in Q-tools (version 3.1.25.604, Q-sense AB, Västra Frölunda, Sweden) on 11 raw data sets of DOPG:DOPC membranes to evaluate SLB area mass using the one-layer Voigt viscoelastic model in comparison to Sauerbrey model on frequency and dissipation data from the 9th, 11th, and 13th harmonics. We used fixed parameters for the fluid density (1000 kg/m^3^), the fluid viscosity (0.001 kg/ms), and the layer density (1000 kg/m^3^). And fitted the layer parameters within following boundaries: viscosity (0.0005–0.01 kg/ms), shear (500 to 1 × 10^9^ Pa), and thickness (1 × 10^−10^ to 1 × 10^−6^ m).

## Results

### Antimicrobial activity of isoeugenol

We assessed the influence of isoeugenol at different concentrations on growth and cell viability of *E. coli* and *L. innocua* cells using turbidity measurements and plate spreading, respectively.

**Figure 2:**
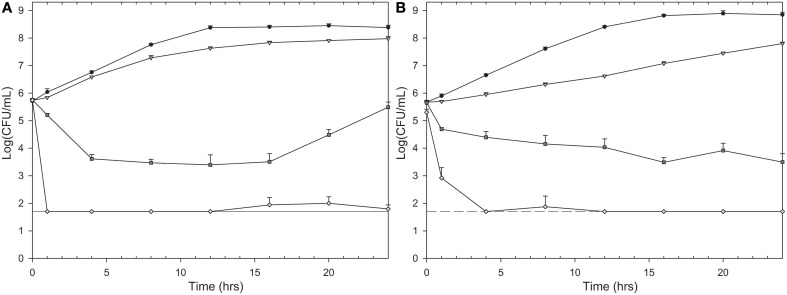
**Time-kill curves of isoeugenol's effect on ***E. coli*** (A) and ***L. innocua*** (B) cell viability**. Cells were treated with isoeugenol at 0 (black circles), ½ × MIC (gray triangle), MIC (dark gray square), or 2× MIC (white diamond) in TSB (pH 6.0) at 25°C. Dashed line indicates detection limit (50 CFU/mL). Error bars are standard deviation (SD) of triplicates.

Isoeugenol inhibited growth of *E. coli* cells at 0.6 mg/mL and *L. innocua* cells at 1 mg/mL, indicating their respective MIC values. Microorganisms treated with isoeugenol at ½ × MIC had a decreased growth rate and decreased maximum growth (Figure 2). Isoeugenol at MIC or 2× MIC initially decreased cell viability, but after 16 h of treatment, *E. coli* cells treated at MIC regained growth, suggesting that isoeugenol exhibit a reversible inhibitory activity against *E. coli* cells (Figure 2A). Interestingly, isoeugenol had an initial bactericidal activity against *L. innocua* that declined in rate after an hour at MIC, whereas 2× MIC killed *L. innocua* cells to undetectable levels (Figure 2B). Based on the time-kill curves, we chose to use 1 h of isoeugenol treatment of bacteria for the remainder of the experiments.

### Isoeugenol does not affect cell morphology

**Figure 3:**
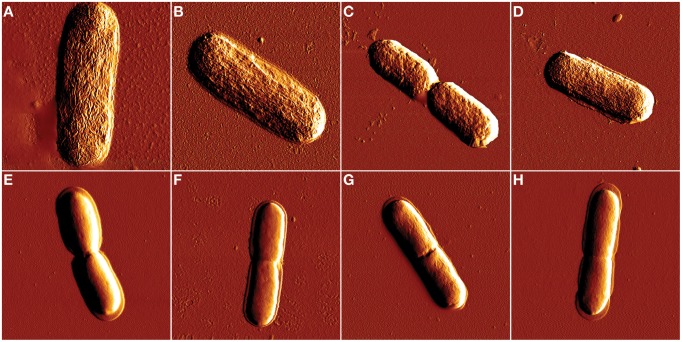
**Atomic force microscopy amplitude images of ***E. coli*** (A–D) and ***L. innocua*** (E–H) cell morphology after isoeugenol treatment**. Cell morphology was imaged in air after treatment with isoeugenol at 0 **(A,E)**, ½ × MIC **(B,F)**, MIC **(C,G)**, or 2× MIC **(D,H)** in MES-buffer (pH = 6.0) at 25°C for an hour. Images of *E. coli* and *L. innocua* were 4 × 4 and 3 × 3 μm, respectively.

After confirming isoeugenol's antimicrobial activity against *E. coli* and *L. innocua* we used AFM to visualize if isoeugenol caused changes in cell morphology. AFM images did not reveal any effect of isoeugenol on cell morphology of neither *E. coli* nor *L. innocua* (Figure 3). No apparent difference in cell length or width other than natural variation of cells in the exponential growth phase existed. Phase images revealed no changes in tip-sample interactions between treated and untreated cells (data not shown).

### Isoeugenol affects membrane integrity and possibly also enzyme activity

**Figure 4:**
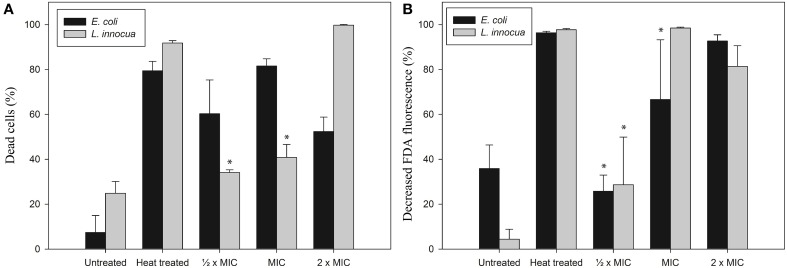
**Isoeugenol induce changes in cytoplasmic membrane integrity (A) and esterase activity (B) of ***E. coli*** (black bars) and ***L. innocua*** (gray bars) cells**. Living and heat treated cells suspended in MES-buffer (pH 6.0) were treated with or without isoeugenol at different concentrations for an hour at 25°C, then harvested and resuspended in MES-buffer together with FDA, followed by incubation for 30 min at 37°C. Cells were harvested and resuspended in MES-buffer before PI staining, except a control with no-stain. Cells with damaged membranes **(A)** or inactive esterases **(B)** were stained with PI or FDA, respectively. Asterisk indicates no statistical significant difference from untreated sample. Error bars = SD (*n* = 3).

Based on the cell morphology results we decided to investigate isoeugenol's effect on cell membrane integrity and intracellular esterase activity using flow cytometry. Isoeugenol compromised the cell membrane of *E. coli* and *L. innocua* cells, which at the same time caused inactivation of intracellular esterase activity (Figure 4).

Isoeugenol affected membrane permeability for *E. coli* cells as indicated by PI staining, and the fraction of cells with damaged membrane increased as the isoeugenol concentration increased from ½ × MIC to MIC. Surprisingly, increasing the concentration further to 2× MIC led to a decrease in the number of *E. coli* cells with a damaged membrane (Figure 4A). Lack of FDA staining is indicative of inactive esterases and/or loss of membrane integrity, and this assay only found an effect from incubation with 2× MIC (Figure 4B). Examination of *L. innocua* showed a trend toward an increasing effect with increasing isoeugenol concentration in both assays, but the effect was not statistically significant at ½ × MIC (Figure 4). These results indicate that isoeugenol permeabilizes bacterial membranes, and possibly inactivates enzymes within *L. innocua* cells prior to membrane permeabilization.

### Isoeugenol affects membrane stability in a non-disruptive manner

The complexity of microorganisms made it difficult to assess isoeugenol's interaction with the cytoplasmic membranes, and therefore we used biophysical investigations with LUVs, GUVs, and SLBs to better understand the mechanism leading to membrane permeabilization. We studied isoeugenol's efficiency for permeabilizing membranes using calcein-encapsulated LUVs, and visualized the direct physical interaction between isoeugenol and membranes following morphology changes of GUVs in suspension, and topographical changes of SLBs on a substrate.

**Figure 5:**
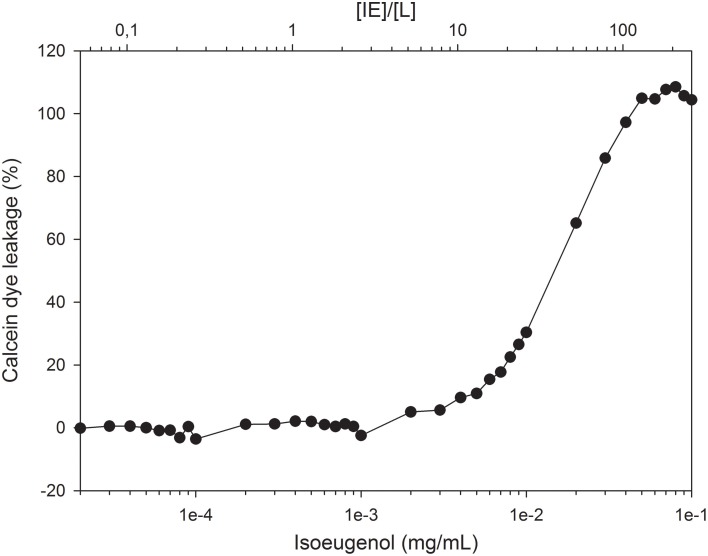
**Increasing concentrations of isoeugenol induce calcein leakage from large unilamellar vesicles of ***E. coli*** polar lipid extract**. Vesicles with encapsulated calcein were suspended in MES-buffer (pH 6.0) and exposed to increasing concentrations of isoeugenol, either expressed as concentration (mg/mL, lower axis) or as a function of isoeugenol to lipid concentration ([IE]/[L], upper axis). Calcein intensity signal was measured before and after 1 h treatment at 37°C, and normalized to the signal obtained at full permeabilization for individual [IE]/[L] ratios. A lipid concentration of ~12 nmol was used for all [IE]/[L] ratios.

The efficiency of isoeugenol to permeabilize phospholipid membranes was investigated using fluorescent dye-encapsulated LUVs. Isoeugenol permeabilized calcein-encapsulated *E. coli* lipid vesicles in a concentration-dependent manner, and required 0.004 mg/mL of isoeugenol (10 molecules of isoeugenol per lipid) to affect membrane permeability, and 0.05 mg/mL isoeugenol (120 molecules of isoeugenol per lipid) for full release (Figure 5).

**Figure 6:**
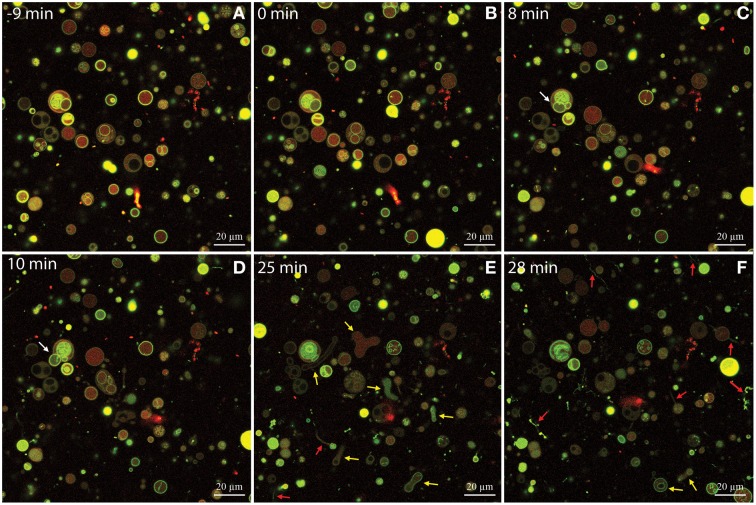
**Time-series of DOPG:DOPC (20:80) giant unilamellar vesicles (GUVs) before and during treatment with isoeugenol**. Confocal laser scanning microscope images were sequentially acquired of the same field-of-view of GUVs stained with the membrane-bound dye Alexa 488 dextran (green) and the intravesicular water-soluble dye Alexa Fluor 633 hydrazide (red). Untreated GUVs **(A)** remained stable and intact until isoeugenol came in contact with GUVs **(B)**. **(C–F)** At later time-points the interaction between isoeugenol and GUVs results in four distinct structural changes of vesicles: Fluctuation of vesicles (not indicated in images), release of intravesicular GUVs (white arrows), affected GUV shape with diverse outcomes (yellow arrows), and tubular or bead protrusions from GUVs (red arrows). Bars correspond to 20 μm.

The use of confocal laser scanning microscopy and DOPG:DOPC GUVs allowed us to visualize the permeabilization of isoeugenol in solution at 0.16 mg/mL, and we observed three interesting details: Firstly, isoeugenol initially induced fluctuation of spherical GUVs without compromising vesicle integrity (Figure 6 and Supporting Video). GUVs released intravesicular GUVs during fluctuation, while maintaining their overall shape and continuity (Figure 6, white arrows, and Supporting Video). Secondly, transformation processes changed GUV's shape into elongated or dumbbell shaped vesicles, which either divided into smaller GUVs, or in some cases instantly fused GUV-ends together forming invaginated sphero-stomatocytes that encapsulated exo-vesicular solution (Figure 6, yellow arrows, and Supporting Video). Lastly, some of the fluctuating GUVs formed tubular protrusions, which transformed into long chains of beads (Figure 6, red arrows, and Supporting Video). Very few GUVs burst open, but when they did the remaining lipid formed long chains of lipid beads or reshaped into new GUVs with beaded protrusions (Supporting video). When isoeugenol reached the GUVs in the field of view, most of the interactions happened within a couple of minutes and continued to affect GUVs for the reminder of the time-series (Figure 6 and supporting video). Interestingly, some GUVs contained intravesicular liposomes that started having morphological changes without disintegration of the outer bilayer, indicative of isoeugenol's ability to permeabilize membranes without affecting membrane continuity (Figure 6 and supporting video). These results suggest that isoeugenol directly affects membrane structure, fluidity, and stability.

**Figure 7:**
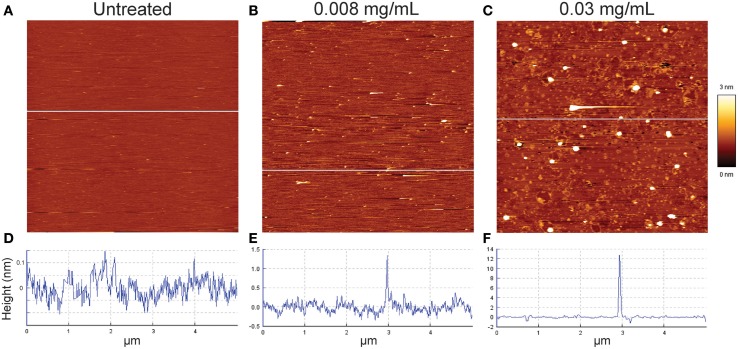
**Contact mode atomic force microscope height images of *E. coli* supported lipid bilayers before (A), and after isoeugenol treatment (B,C)**. Untreated *E. coli* lipid **(A,D)** was imaged in MES-buffer (pH 6.0) at 37°C, and subsequently treated with either low **(B,E)** or high **(C,F)** concentration isoeugenol as evaluated on calcein leakage (Figure 5) for an hour and rinsed with fresh MES-buffer before imaging. Cross-sectional profiles **(D–F)** taken at the white line in the corresponding height images have different height scales. Images are 5 × 5 μm segments, where the bright and dark areas correspond to higher or lower structures than the mean average height of the bilayer.

In order to visualize the permeabilization mechanism in more detail, we monitored how isoeugenol affected the topography of SLBs using AFM. We exposed SLBs to 0.008 or 0.03 mg/mL of isoeugenol, which induced low and high leakage from calcein-encapsulated vesicles, respectively. Untreated *E. coli* polar SLBs topography appeared homogeneous and intact with small height differences of approximately 0.2 nm (Figure 7A). Low isoeugenol concentrations induced the appearance of small protrusions from the SLB with a height ranging between 1 and 2.5 nm above the SLB (Figure 7B). At high isoeugenol concentration, the membrane topography became more heterogeneously distributed, with small membrane indentations and 12 nm large “bubbles” protruding from the membrane (Figure 7C). This indicates that isoeugenol affects membrane topography, resulting in changes in lipid packing and/or formation of transient pores, which disturbs membrane stability.

### Isoeugenol interacts reversible with membranes making bilayers more viscoelastic

Preceding results indicated that nonionic isoeugenol molecules could situate in the membrane and change the permeability, topography, and shape dynamics of membranes. In order to assess the effect of isoeugenol on membrane fluidity and viscoelastic properties, we measured fluorescence anisotropy of DPH-probed LUVs and monitored the real-time interaction dynamics between isoeugenol and membranes using QCM-D.

**Figure 8:**
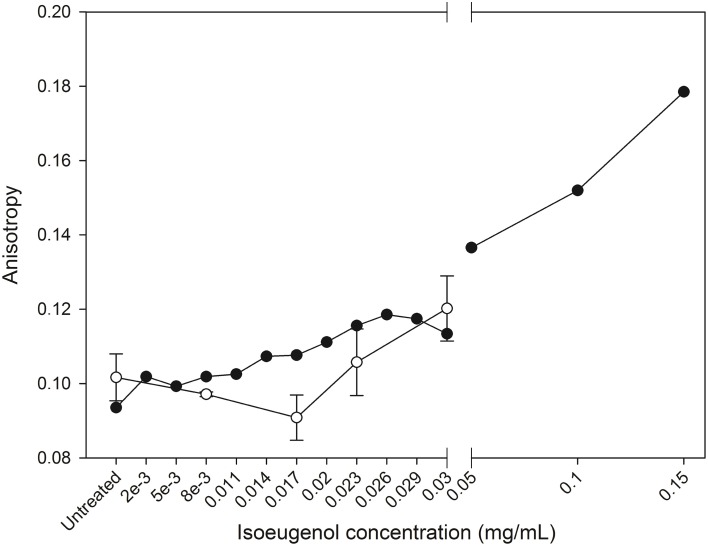
**Isoeugenol's effect on large unilamellar vesicles fluidity**. Fluorescence anisotropy of diphenylhexatriene in vesicles composed of DOPG:DOPC (20:80) (black circles) or *E. coli* polar lipid extract (white circles) were measured as a function of increasing concentrations of isoeugenol in MES-buffer (pH 6.0) at 25 or 37°C, respectively. Anisotropy is a measure of lipid ordering and bilayer fluidity, and an increased fluidity have a decrease in anisotropy, while low membrane fluidity has increases anisotropy signal. Mean anisotropy values are shown for duplicates of DOPG:DOPC (20:80) vesicles, while average values for *E. coli* vesicles are shown for triplicates. Error bars = SD (*n* = 3).

Surprisingly, treatment of DPH-probed DOPG:DOPC lipid vesicles with increasing concentrations of isoeugenol for 5 min indicated an increase in anisotropy values compared to untreated DPH-vesicles, demonstrating isoeugenol decreased membrane fluidity at room temperature (Figure 8). DPH-probed *E. coli* lipid at 37°C treated with 0.03 mg/mL isoeugenol had increased DPH fluorescence anisotropy values that exceeded the signal of untreated DPH-vesicles (*p* < 0.05) (Figure 8). These results can be interpreted as isoeugenol increases the acyl chain order of vesicle lipid bilayers, thus exhibiting a membrane rigidifying effect.

**Figure 9:**
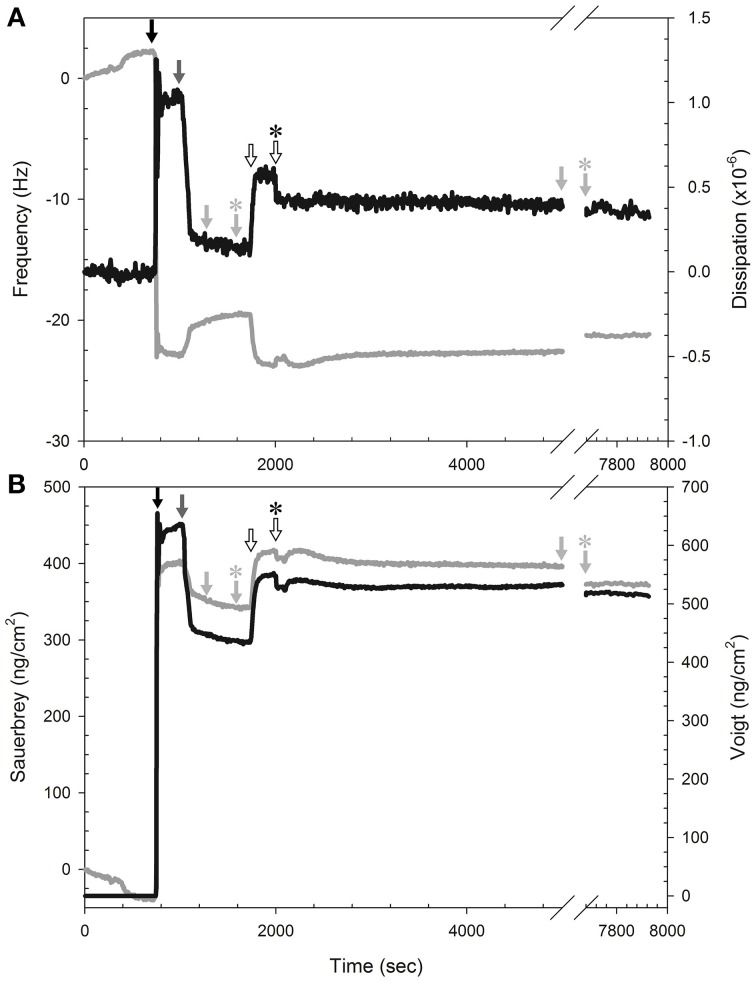
**Isoeugenol binds reversible to SLB and increases membrane hydration. (A)** Representative measurement of frequency (gray line) and dissipation (black line) changes at the 9th harmonic of a DOPG:DOPC (20:80) membrane at 37°C. To form SLBs we introduced lipid vesicles (black arrow) in MES-buffer (pH 6.0), followed by pure MES-buffer (pH 6.0) with 20 mM CaCl_2_ (dark gray arrow). All rinsing steps was performed with fresh MES-buffer without CaCl_2_ (pH 6.0, light gray arrow) to remove excess lipid, and the flow was stopped momentarily (light gray arrow with asterisk). Isoeugenol was injected at a final concentration of 0.03 mg/mL (hollow arrow) until chamber content was exchanged, and the flow was stopped (hollow arrow with asterisk) until treatment had lasted 60 min, followed by buffer-rinse for 45 min before the rinsing was stopped (light gray arrow with asterisk). **(B)** Modeling of area mass changes during SLB formation and isoeugenol exposure using Sauerbrey (gray line) and one-layer Voigt (black line).

We evaluated isoeugenol's effect at 0.008 and 0.03 mg/mL on the mass and viscosity of SLBs consisting of 20:80 DOPG:DOPC in real-time on silica surfaces using the QCM-D technique. The QCM-D profiles for all SLBs showed a significant baseline drift of the frequency, not dissipation, just prior to addition of the SUVs (Figure 9A), thus the data were only analyzed semi-quantitatively by comparing trends in frequency shift, Δ*f*, and dissipation, Δ*D*. The creation of SLBs (*n* = 11) resulted in frequency shifts of −20.0 ± 2.4 Hz. A concomitant low dissipation value of 0.26 ± 0.09 × 10^−6^ indicated formation of a stable DOPG:DOPC bilayer (Figure 9A). Addition of isoeugenol at 0.008 mg/mL resulted in no noticeable changes. However, addition of 0.03 mg/mL resulted in a drop in frequency after rinsing, suggesting that isoeugenol binds to membranes and thus increasing the mass of the SBL (Figure 9A). The corresponding dissipation of SLBs increased during and after treatment with isoeugenol, and the higher dissipation per frequency could suggest a more hydrated SLB, which is less rigid or more saturated with fluid after exposure to isoeugenol (Wang et al., 2011).

Analysing the QCM-D data using the Sauerbrey and Voigt model, respectively, confirmed that isoeugenol bound to the SLB as we observed an increase in area mass after exposure to 0.03 mg/mL isoeugenol (Figure 9B). Interestingly, the area mass of the membrane exposed to high concentration of isoeugenol remained constant during the exposure, and after the final rinse the mass dropped but remained above the initial SLB, this suggests that isoeugenol interact with membranes in a reversible manner (Figure 9B). Collectively, the QCM-D results suggest that a small fraction of isoeugenol bind to bilayers making it more viscoelastic (more hydrated).

## Discussion

### Antimicrobial activity of isoeugenol

We assessed isoeugenol's inhibitory activity and its effect on cell viability of *E. coli* and *L. innocua*. The MIC values for *E. coli* were between one and six-fold higher than previous reports (Zemek et al., 1979; Kang et al., 1992). Isoeugenol's MIC against *L. innocua* was five-fold higher than previously determined for *L. innocua* and the closely related *Listeria monocytogenes* (Faith et al., 1992; Vitt et al., 2001). The discrepancy between our and previous studies could be caused by different cultivation conditions, use of different strains which may show different susceptibility, or the use of different agents, such as ethanol, dimethyl sulphoxide, dimethylformamide, or Tween 80, to disperse isoeugenol in solution (Faith et al., 1992; Kang et al., 1992; Vitt et al., 2001).

Time-kill curves provided more insight into isoeugenol's effect on viability and growth over time. Although growth of *E. coli* was initially inhibited and the number of viable cells even dropped by 1–2 log units when adding isoeugenol at the MIC concentration, growth resumed after approximately 16 h of incubation. Analyzing the result from a MIC test after 24 h incubation resulted in a MIC value of 0.6 mg/mL, but if the culture had been incubated longer we would have measured net growth at this concentration. This observation underlines the sensitivity of the MIC assay regarding duration and conditions of incubation. The regrowth of *E. coli* could be caused by the following; (i) isoeugenol is a volatile compound and could have evaporated, allowing surviving cells to grow when the concentration fell beneath an inhibitory threshold, (ii) cells could adapt to isoeugenol by excreting molecules, or modifying the LPS composition, membrane order, or cell hydrophobicity (Sikkema et al., 1995), or (iii) isoeugenol could be inactivated by immobilization or conversion to less active compound, such as vanillin or vanillic acid, as seen in *Bacillus* spp., and *Pseudomonas* spp. cultures (Shimoni et al., 2000; Furukawa et al., 2003; Kaur and Chakraborty, 2013). Future studies using gas chromatography measurements could be used to conclude which scenario causes regrowth of *E. coli*.

### Isoeugenol destabilizes and permeabilizes membranes

Visual investigation by AFM indicated that isoeugenol had cytoplasmic or intracellular targets because cells underwent no morphological changes to the outer membrane or cell wall (Figure 3). Furthermore, *E. coli* and *L. innocua* cells were fluorescently stained by PI but not FDA, which confirms that the cytoplasmic membrane was damaged and suggests that enzyme activity may also be affected (Figure 4) (Schenk et al., 2011). We observed a discrepancy in our data for *E. coli* exposed to isoeugenol at 2× MIC. PI staining showed permeabilization of all cells at 1 × MIC, and it is therefore counterintuitive that the percentage of PI positive *E. coli* decreased when further increasing the isoeugenol concentration to 2× MIC as we detected no viable cells at this concentration (Figures 2A, 4A). Furthermore, the results from PI- and FDA-staining of *E. coli* at 2× MIC did not agree. PI staining indicated permeabilization of approximately half of the cells, while >90% were FDA-negative. We observed the same disagreement between PI and FDA staining of *L. innocua* at 1 × MIC. It was previously suggested that phenylpropenes such as eugenol, cinnamaldehyde, and vanillin could interact with both membranes and intracellular proteins, thus it is possible that isoeugenol has two targets and inhibited enzyme activity in a larger fraction of cells than those with permeabilized membranes (Fitzgerald et al., 2004; Gill and Holley, 2006a; Di Pasqua et al., 2007; Domadia et al., 2007; Hyldgaard et al., 2012a).

In the following, we argue that isoeugenol situate in membranes and contrary to results from the membrane fluidity experiment with DPH, isoeugenol has a fluidifying effect on the cytoplasmic membrane. Our results demonstrate that isoeugenol interacts with and situates in membranes, evidenced as an increased mass and changed topography of SLBs, which consequently destabilize and become permeable (Figures 5, 7, 9B). Isoeugenol's interaction with membranes could cause changes in membrane topography because of sequestering of lipids with preferences for different curvature or acyl chain lengths (Mrówczyńska et al., 2011; Lichtenberg et al., 2013). Isoeugenol is nonionic so it would not sequester lipids based on different headgroup charges as it requires cationic charged molecules to cluster anionic lipids from zwitterionic ones (Epand et al., 2010). Although we detected decreased membrane fluidity of DPH-probed LUVs, we propose that isoeugenol increases membrane fluidity based on more hydrated bilayers observed by QCM-D upon isoeugenol exposure (Figure 9A). Furthermore, we observed that isoeugenol induced structural changes to GUVs in the form of fluctuations, tubular and bead protrusions, release of intravesicular GUVs and formation of sphero-stomatocytes, which previous studies attributed to increased membrane fluidity in the case of stilbenes, octaethyleneglycol dodecylether, and amyloid beta peptides in the presence of oxysterols (Mavcic et al., 2004; Morita et al., 2010; Phan et al., 2013) (Figure 6 and Supporting Video). Structural changes of GUVs in the presence of isoeugenol therefore contradicts structural changes of GUVs exposed to rigidifying molecules like flavonoids, which cause aggregation, endo-budding, and bursting with subsequent release of daughter vesicles or release of small lumps (Phan et al., 2014). Fluctuations (or quakes) of GUVs are an effect of changes in area-to-volume ratio due to insertion of molecules into membranes, and might cause transient holes in the membrane without affecting GUV shape and continuity, which could explain the leakiness of vesicles and cells when exposed to isoeugenol (Nomura et al., 2001; Hamada et al., 2012). Formation of tubular and bead protrusions relieve this excess area of GUVs, whereas sphero-stomatocytes have been proposed to arise when foreign molecules penetrate deeply into the bilayer thus promoting large positive and negative curvature changes in both membrane leaflets (Phan et al., 2013, 2014). In addition to GUVs, QCM-D measurements confirmed that membranes became more hydrated when isoeugenol incorporated into lipid bilayers (Figure 9A). Isoeugenol's ability to induce structural changes in membranes, like tubular or bead structures, might be more pronounced for LUVs than GUVs and SLBs because of lipid-packing defects caused by increased curvature, which enhances the accessibility of isoeugenol to exposed hydrophobic areas in curved membranes (Huang and Ramamurthi, 2010). It was previously demonstrated that cholesterol induce formation of similar GUV shape changes where “beads” contain ordered lipid, while the nodes contain disordered lipid (Yanagisawa et al., 2010). Hence, we speculate that the decreased membrane fluidity measured for LUVs could be an artifact caused by formation of tubular or bead structures (Figure 6). In our case the bead-like structures might restrict the movement of DPH within the hydrophobic region of vesicles, while fluid membrane will situate at nodes in-between beads (Yanagisawa et al., 2010). This can explain the contradiction between DPH results, and QCM-D and GUV results.

### Isoeugenol's mode of action resembles a non-disruptive detergent-like mechanism

In this section we hypothesize that isoeugenol's mechanism of action on cytoplasmic membranes resembles a non-disruptive detergent-like mechanism. Isoeugenol is a small nonionic molecule with an aromatic ring and a hydroxyl-group, which has a structural resemblance to e.g., the aromatic amino acid tyrosine or perillaldehyde, both having the ability to localize at the lipid headgroup region and thus affect packing of lipids (Sanderson and Whelan, 2004; Sanderson, 2005; Witzke et al., 2010). Based on molecular dynamic simulations of structurally related compounds, we propose that isoeugenol form H-bonds between the polar side chains and phosphate or carbonyl groups on phospholipid headgroups, and position around 1 nm from the center of the bilayer with the propenyl-chain intercalating into the acyl chains to destabilize membranes (Sanderson and Whelan, 2004; Witzke et al., 2010; Lomize et al., 2011). Isoeugenol would thus take up space in the head group region of the outer leaflet of membranes, increase membrane fluidity, and enhance the interfacial lateral pressure of the outer leaflet of the membrane, which in turn affects lipid packing and destabilize membranes to become leaky and induce shape changes.

Molecular dynamics simulations have shown that essential oil constituents like perillaldehyde or perillyl alcohol carrying more hydrophilic groups require more energy to traverse the membrane and have a 700-fold slower flip-flop rate than the nonpolar constituent limonene (Witzke et al., 2010). The low flip-flop rate could make it difficult for the essential oil constituents to diffuse across membranes and interact with intracellular targets without permeabilizing the membrane. In accordance with structural changes of GUVs, isoeugenol's destabilizing effect resembles a detergent-like mechanism for slow flip-flop rate sodium dodecyl sulfate or slow addition of the nonionic detergent octaethyleneglycol dodecylether, however isoeugenol differed from these detergents by not causing liposomal shrinkage or vesicle bursting (Mavcic et al., 2004; Sudbrack et al., 2010; Lichtenberg et al., 2013) (Figure 7 and Supporting Video 1). Furthermore, isoeugenol molecules might interact with membrane-bound proteins like ATPase in a similar manner as observed for eugenol (Gill and Holley, 2006b), thus affecting a cell's ability to maintain homeostasis.

In conclusion, we propose that isoeugenol interacts reversibly with *E. coli* membranes through a non-disruptive detergent-like mechanism that destabilizes membranes to become leaky. Because of isoeugenol's hydrophobic nature, it has tendency to interact with both neutral and charged membranes, which is also indicative for its broad antimicrobial range as it inhibits both yeast and bacteria (Kang et al., 1992). Differences in susceptibility of various microorganisms could be caused by different efficiencies of efflux pump systems (Nikaido, 1996). Future studies could focus on investigating isoeugenol's intracellular effect on different proteins and enzymes as the structural isomer, eugenol, has been shown to inhibit a myriad of different enzymes such as ATPase, histidine decarboxylase, amylase, and protease (Thoroski et al., 1989; Wendakoon and Morihiko, 1995; Gill and Holley, 2006b). Detailed knowledge about how isoeugenol gains entry to the cytoplasmic membrane in Gram-negative bacteria could be obtained by studying isoeugenol's interaction with LPS and porins. The cytoplasmic membrane of Gram-negative bacteria mainly consists of zwitterionic phospholipids; 5–20% CL, 5–21% PG, and 60–80% PE, while the majority of phospholipids in Gram-positive bacteria are anionic phospholipids; 15–50% CL, 40–70% PG, and 0–40 PE (Epand and Epand, 2011), or special phospholipids like lysyl-PG and lysyl-CL found in *L. innocua* (Fischer and Leopold, 1999). Thus, we suggest investigating how isoeugenol interacts with cytoplasmic membranes of different composition, acyl chain lengths, or membrane curvature to further understand how isoeugenol affect the membrane of *L. innocua* (Hatzakis et al., 2009; Tomita et al., 2011). This knowledge could contribute to a better understanding of the biological differences that lead to variable susceptibility.

### Conflict of interest statement

The work was co-funded by DuPont Nutrition Biosciences and the Danish Innovation Fund as part of the Industrial Ph.D. programme. DuPont has not made any changes to how the results obtained in the project were disseminated.

## References

[B1] Balhara V. Schmidt R. Gorr S.-U. Dewolf C. (2013). Membrane selectivity and biophysical studies of the antimicrobial peptide GL13K. Biochim. Biophys. Acta 1828, 2193–2203. 10.1016/j.bbamem.2013.05.027 23747365

[B2] Bhatia R. Shreaz S. Khan N. Muralidhar S. Basir S. F. Manzoor N.. (2012). Proton pumping ATPase mediated fungicidal activity of two essential oil components. J. Basic Microbiol. 52, 504–512. 10.1002/jobm.201100272 22143929

[B3] Brenes A. Roura E. (2010). Essential oils in poultry nutrition: main effects and modes of action. Anim. Feed Sci. Technol. 158, 1–14. 10.1016/j.anifeedsci.2010.03.007

[B4] Burt S. (2004). Essential oils: their antibacterial properties and potential applications in foods - a review. Int. J. Food Microbiol. 94, 223–253. 10.1016/j.ijfoodmicro.2004.03.022 15246235

[B5] Dal Pozzo M. Loreto É. S. Santurio D. F. Alves S. H. Rossatto L. De Vargas A. C. (2012). Antibacterial activity of essential oil of cinnamon and trans-cinnamaldehyde against *Staphylococcus* spp. Isolated from clinical mastitis of cattle and goats. Acta Sci. Vet. 40, 1080.

[B6] Desbois A. P. Smith V. J. (2010). Antibacterial free fatty acids: activities, mechanisms of action and biotechnological potential. Appl. Microbiol. Biotechnol. 85, 1629–1642. 10.1007/s00253-009-2355-3 19956944

[B7] Di Pasqua R. Betts G. Hoskins N. Edwards M. Ercolini D. Mauriello G. (2007). Membrane toxicity of antimicrobial compounds from essential oils. J. Agric. Food Chem. 55, 4863–4870. 10.1021/jf0636465 17497876

[B8] Domadia P. Swarup S. Bhunia A. Sivaraman J. Dasgupta D. (2007). Inhibition of bacterial cell division protein FtsZ by cinnamaldehyde. Biochem. Pharmacol. 74, 831–840. 10.1016/j.bcp.2007.06.029 17662960

[B9] Epand R. F. Maloy W. L. Ramamoorthy A. Epand R. M. (2010). Probing the “charge cluster mechanism” in amphipathic helical cationic antimicrobial peptides. Biochemistry 49, 4076–4084. 10.1021/bi100378m 20387900 PMC2868066

[B10] Epand R. M. Epand R. F. (2011). Bacterial membrane lipids in the action of antimicrobial agents. J. Pept. Sci. 17, 298–305. 10.1002/psc.1319 21480436

[B11] Faith N. G. Yousef A. E. Luchansky J. B. (1992). Inhibition of *Listeria monocytogenes* by liquid smoke and isoeugenol, a phenolic component found in smoke. J. Food Saf. 12, 303–314. 10.1111/j.1745-4565.1992.tb00086.x

[B12] Fischer W. Leopold K. (1999). Polar lipids of four Listeria species containing L-lysylcardiolipin, a novel lipid structure, and other unique phospholipids. Int. J. Syst. Bacteriol. 49, 653–662. 10.1099/00207713-49-2-653 10408878

[B13] Fitzgerald D. J. Stratford M. Gasson M. J. Ueckert J. Bos A. Narbad A. (2004). Mode of antimicrobial of vanillin against *Escherichia coli, Lactobacillus plantarum* and *Listeria innocua*. J. Appl. Microbiol. 97, 104–113. 10.1111/j.1365-2672.2004.02275.x 15186447

[B14] Furukawa H. Morita H. Yoshida T. Nagasawa T. (2003). Conversion of isoeugenol into vanillic acid by *Pseudomonas putida* I58 cells exhibiting high isoeugenol-degrading activity. J. Biosci. Bioeng. 96, 401–403. 10.1016/S1389-1723(03)90145-9 16233545

[B15] Gill A. O. Holley R. A. (2006a). Disruption of *Escherichia coli, Listeria monocytogenes* and *Lactobacillus sakei* cellular membranes by plant oil aromatics. Int. J. Food Microbiol. 108, 1–9. 10.1016/j.ijfoodmicro.2005.10.009 16417936

[B16] Gill A. O. Holley R. A. (2006b). Inhibition of membrane bound ATPases of *Escherichia coli* and *Listeria monocytogenes* by plant oil aromatics. Int. J. Food Microbiol. 111, 170–174. 10.1016/j.ijfoodmicro.2006.04.046 16828188

[B17] Hamada T. Hagihara H. Morita M. Vestergaard M. D. C. Tsujino Y. Takagi M. (2012). Physicochemical profiling of surfactant-induced membrane dynamics in a cell-sized liposome. J. Phys. Chem. Lett. 3, 430–435. 10.1021/jz2016044 26285862

[B18] Hatzakis N. S. Bhatia V. K. Larsen J. Madsen K. L. Bolinger P.-Y. Kunding A. H.. (2009). How curved membranes recruit amphipathic helices and protein anchoring motifs. Nat. Chem. Biol. 5, 835–841. 10.1038/nchembio.213 19749743

[B19] Hemaiswarya S. Doble M. (2009). Synergistic interaction of eugenol with antibiotics against Gram negative bacteria. Phytomedicine 16, 997–1005. 10.1016/j.phymed.2009.04.006 19540744

[B20] Huang K. C. Ramamurthi K. S. (2010). Macromolecules that prefer their membranes curvy: MicroReview. Mol. Microbiol. 76, 822–832. 10.1111/j.1365-2958.2010.07168.x 20444099 PMC2909655

[B21] Hyldgaard M. Mygind T. Meyer R. L. (2012a). Essential oils in food preservation: mode of action, synergies, and interactions with food matrix components. Front. Microbiol. 3:12. 10.3389/fmicb.2012.00012 22291693 PMC3265747

[B22] Hyldgaard M. Sutherland D. S. Sundh M. Mygind T. Meyer R. L. (2012b). Antimicrobial mechanism of monocaprylate. Appl. Environ. Microbiol. 78, 2957–2965. 10.1128/AEM.07224-11 22344642 PMC3318790

[B23] Janssens J. Laekeman G. M. Pieters L. A. Totte J. Herman A. G. Vlietinck A. J. (1990). Nutmeg oil: identification and quantitation of its most active constituents as inhibitors of platelet aggregation. J. Ethnopharmacol. 29, 179–188. 10.1016/0378-8741(90)90054-W 2115612

[B24] Kang R. Helms R. Stout M. J. Jaber H. Chen Z. Nakatsu T. (1992). Antimicrobial activity of the volatile constituents of *Perilla frutescens* and its synergistic effects with polygodial. J. Agric. Food Chem. 40, 2328–2330. 10.1021/jf00023a054

[B25] Kaur B. Chakraborty D. (2013). Biotechnological and molecular approaches for vanillin production: a review. Appl. Biochem. Biotechnol. 169, 1353–1372. 10.1007/s12010-012-0066-1 23306890

[B26] Laekeman G. M. Van Hoof L. Haemers A. Berghe D. A. V. Herman A. G. Vlietinck A. J. (1990). Eugenol a valuable compound for *in vitro* experimental research and worthwhile for further *in vivo* investigation. Phytother. Res. 4, 90–96. 10.1002/ptr.2650040304

[B27] Lichtenberg D. Ahyayauch H. Goñi F. M. (2013). The mechanism of detergent solubilization of lipid bilayers. Biophys. J. 105, 289–299. 10.1016/j.bpj.2013.06.007 23870250 PMC3714928

[B28] Lomize A. L. Pogozheva I. D. Mosberg H. I. (2011). Anisotropic solvent model of the lipid bilayer. 2. Energetics of insertion of small molecules, peptides, and proteins in membranes. J. Chem. Inf. Model. 51, 930–946. 10.1021/ci200020k 21438606 PMC3091260

[B29] Mavcic B. Babnik B. Iglic A. Kanduser M. Slivnik T. Kralj-Iglic V. (2004). Shape transformation of giant phospholipid vesicles at high concentrations of C12E8. Bioelectrochemistry 63, 183–188. 10.1016/j.bioelechem.2003.09.022 15110270

[B30] Meyer R. L. Zhou X. Tang L. Arpanaei A. Kingshott P. Besenbacher F. (2010). Immobilisation of living bacteria for AFM imaging under physiological conditions. Ultramicroscopy 110, 1349–1357. 10.1016/j.ultramic.2010.06.010 20619542

[B31] Miles A. A. Misra S. S. Irwin J. O. (1938). The estimation of the bactericidal power of the blood. Epidemiol. Infect. 38, 732–749. 10.1017/s002217240001158x 20475467 PMC2199673

[B32] Morita M. Vestergaard M. D. Hamada T. Takagi M. (2010). Real-time observation of model membrane dynamics induced by Alzheimer's amyloid beta. Biophys. Chem. 147, 81–86. 10.1016/j.bpc.2009.12.004 20060637

[B33] Mrówczyńska L. Salzer U. Iglič A. Hägerstrand H. (2011). Curvature factor and membrane solubilization, with particular reference to membrane rafts. Cell Biol. Int. 35, 991–995. 10.1042/CBI20100786 21438858

[B34] Nikaido H. (1996). Multidrug efflux pumps of gram-negative bacteria. J. Bacteriol. 178, 5853–5859. 8830678 10.1128/jb.178.20.5853-5859.1996 PMC178438

[B35] Nomura F. Nagata M. Inaba T. Hiramatsu H. Hotani H. Takiguchi K. (2001). Capabilities of liposomes for topological transformation. Proc. Natl. Acad. Sci. U.S.A. 98, 2340–2345. 10.1073/pnas.041419098 11226241 PMC30140

[B36] Phan H. T. T. Hata T. Morita M. Yoda T. Hamada T. Vestergaard M. C.. (2013). The effect of oxysterols on the interaction of Alzheimer's amyloid beta with model membranes. Biochim. Biophys. Acta 1828, 2487–2495. 10.1016/j.bbamem.2013.06.021 23800382

[B37] Phan H. T. T. Yoda T. Chahal B. Morita M. Takagi M. Vestergaard M. D. C. (2014). Structure-dependent interactions of polyphenols with a biomimetic membrane system. Biochim. Biophys. Acta 1838, 2670–2677. 10.1016/j.bbamem.2014.07.001 25016053

[B38] Rajakumar D. V. Rao M. N. A. (1993). Dehydrozingerone and isoeugenol as inhibitors of lipid peroxidation and as free radical scavengers. Biochem. Pharmacol. 46, 2067–2072. 10.1016/0006-2952(93)90649-H 8267655

[B39] Sanderson J. M. (2005). Peptide-lipid interactions: insights and perspectives. Org. Biomol. Chem. 3, 201–212. 10.1039/b415499a 15632958

[B40] Sanderson J. M. Whelan E. J. (2004). Characterisation of the interactions of aromatic amino acids with diacetyl phosphatidylcholine. Phys. Chem. Chem. Phys. 6, 1012–1017. 10.1039/b312184d

[B41] Schenk M. Raffellini S. Guerrero S. Blanco G. A. Alzamora S. M. (2011). Inactivation of *Escherichia coli, Listeria innocua* and *Saccharomyces cerevisiae* by UV-C light: study of cell injury by flow cytometry. LWT Food Sci. Technol. 44, 191–198. 10.1016/j.lwt.2010.05.012

[B42] Shaw J. E. Alattia J. R. Verity J. E. Privé G. G. Yip C. M. (2006). Mechanisms of antimicrobial peptide action: studies of indolicidin assembly at model membrane interfaces by *in situ* atomic force microscopy. J. Struct. Biol. 154, 42–58. 10.1016/j.jsb.2005.11.016 16459101

[B43] Shimoni E. Ravid U. Shoham Y. (2000). Isolation of a *Bacillus* sp. capable of transforming isoeugenol to vanillin. J. Biotechnol. 78, 1–9. 10.1016/S0168-1656(99)00199-6 10702906

[B44] Sikkema J. De Bont J. A. M. Poolman B. (1995). Mechanisms of membrane toxicity of hydrocarbons. Microbiol. Rev. 59, 201–222. 7603409 10.1128/mr.59.2.201-222.1995 PMC239360

[B45] Sudbrack T. P. Archilha N. L. Itri R. Riske K. A. (2010). Observing the solubilization of lipid bilayers by detergents with optical microscopy of GUVs. J. Phys. Chem. B 115, 269–277. 10.1021/jp108653e 21171656

[B46] Thoroski J. Blank G. Biliaderis C. (1989). Eugenol induced inhibition of extracellular enzyme production by *Bacillus cereus*. J. Food Prot. 52, 399–403. 10.4315/0362-028X-52.6.399 31003298

[B47] Tomita T. Sugawara T. Wakamoto Y. (2011). Multitude of morphological dynamics of giant multilamellar vesicles in regulated nonequilibrium environments. Langmuir 27, 10106–10112. 10.1021/la2018456 21702436

[B48] Trevors J. T. (2003). Fluorescent probes for bacterial cytoplasmic membrane research. J. Biochem. Biophys. Methods 57, 87–103. 10.1016/S0165-022X(03)00076-9 12915003

[B49] Vad B. S. Bertelsen K. Johansen C. H. Pedersen J. M. Skrydstrup T. Nielsen N. C.. (2010). Pardaxin permeabilizes vesicles more efficiently by pore formation than by disruption. Biophys. J. 98, 576–585. 10.1016/j.bpj.2009.08.063 20159154 PMC2820650

[B50] Vitt S. M. Himelbloom B. H. Crapo C. A. (2001). Inhibition of *Listeria innocua* and *L. monocytogenes* in a laboratory medium and cold-smoked salmon containing liquid smoke. J. Food Saf. 21, 111–125. 10.1111/j.1745-4565.2001.tb00311.x

[B51] Wang K. F. Nagarajan R. Mello C. M. Camesano T. A. (2011). Characterization of supported lipid bilayer disruption by chrysophsin-3 using QCM-D. J. Phys. Chem. B 115, 15228–15235. 10.1021/jp209658y 22085290

[B52] Wendakoon C. N. Morihiko S. (1995). Inhibition of amino acid decarboxylase activity of *Enterobacter aerogenes* by active components in spices. J. Food Prot. 58, 280–283. 10.4315/0362-028X-58.3.280 31137282

[B53] Witzke S. Duelund L. Kongsted J. Petersen M. Mouritsen O. G. Khandelia H. (2010). Inclusion of terpenoid plant extracts in lipid bilayers investigated by molecular dynamics simulations. J. Phys. Chem. B 114, 15825–15831. 10.1021/jp108675b 21070035

[B54] Yanagisawa M. Imai M. Taniguchi T. (2010). Periodic modulation of tubular vesicles induced by phase separation. Phys. Rev. E 82:051928. 10.1103/PhysRevE.82.051928 21230521

[B55] Yeaman M. R. Yount N. Y. (2003). Mechanisms of antimicrobial peptide action and resistance. Pharmacol. Rev. 55, 27–55. 10.1124/pr.55.1.2 12615953

[B56] Yu L. Guo L. Ding J. L. Ho B. Feng S. S. Popplewell J.. (2009). Interaction of an artificial antimicrobial peptide with lipid membranes. Biochim. Biophys. Acta 1788, 333–344. 10.1016/j.bbamem.2008.10.005 19013127

[B57] Zemek J. Kosikova B. Augustin J. Joniak D. (1979). Antibiotic properties of lignin components. Folia Microbiol. 24, 483–486. 10.1007/BF02927180 389763

[B58] Zemek J. Valent M. Pódová M. Košíková B. Joniak D. (1987). Antimicrobiai properties of aromatic compounds of plant origin. Folia Microbiol. 32, 421–425. 10.1007/bf02887573 3121479

