# Supplementary material for: Isoeugenol has a non-disruptive detergent-like mechanism of action
Source: Front Microbiol. 2015 Jul 28;6:754. doi: 10.3389/fmicb.2015.00754 (PMC4517379; doi:10.3389/fmicb.2015.00754)

***Supplementary Material***

**Isoeugenol has a non-Disruptive Detergent-like Mechanism of Action**

**Morten Hyldgaard^1,2^, Tina Mygind^2^, Roxana Piotrowska^3^, Morten Foss^3^, and Rikke L. Meyer^1*^**

^1^Biofilm Group, Interdisciplinary Nanoscience Center, Aarhus University, Aarhus, Denmark ^2^Antimicrobials and Antioxidants, Nutrition and Health, DuPont Nutrition Biosciences, Brabrand, Denmark

^3^Biomedical Surface Group, Interdisciplinary Nanoscience Center, Aarhus University, Aarhus, Denmark

*** Correspondence:** Dr. Rikke Louise Meyer, Biofilm Group, Interdisciplinary Nanoscience Center, Aarhus University, Gustav Wieds Vej 14, Aarhus C, 8000, Denmark.

Rikke.meyer@inano.au.dk

1. **Supplementary Data**

**Supplementary video. Confocal laser scanning microscope time-series of giant unilamellar vesicles (GUVs).** Fluorescence images of the progressive response of GUVs stained with Alexa 633 and Alexa 488 in 200 mM glucose solution to isoeugenol at room temperature. Images were acquired at the same field-of-view for 38 minutes, and isoeugenol reaches the imaged GUVs after approximately 9 minutes after onset of the experiment. The scale bar is 20 µm.


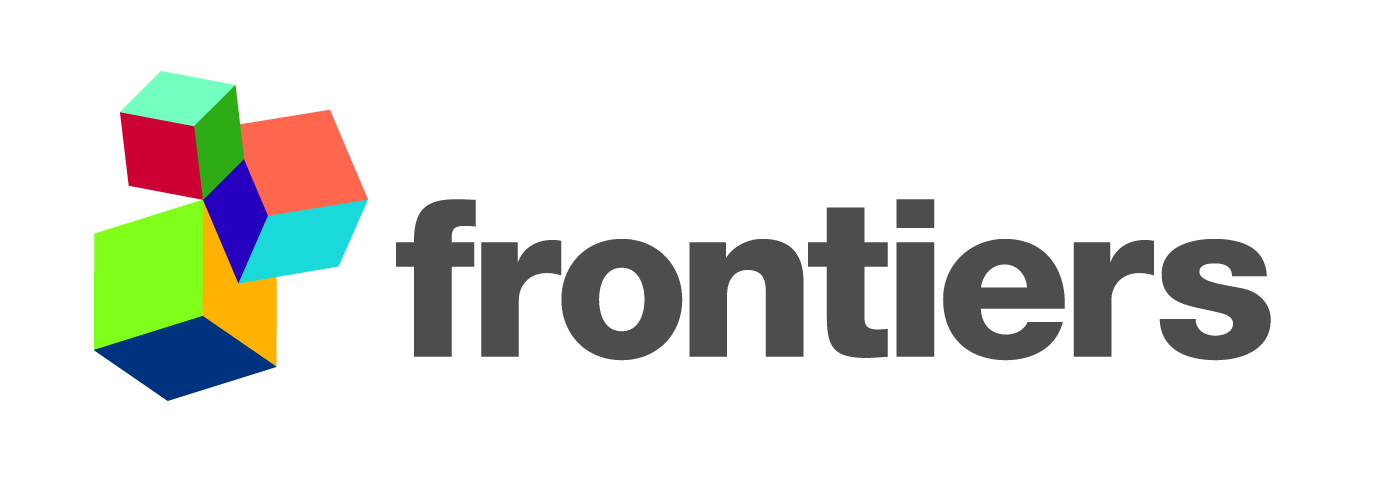

Supplement: Supplementary file 2 [file DataSheet1.DOCX]
